# Association between handgrip strength, walking, age-related illnesses and cognitive status in a sample of Portuguese centenarians

**DOI:** 10.1186/s11556-017-0178-2

**Published:** 2017-07-01

**Authors:** Maria Vaz-Patto, Belén Bueno, Óscar Ribeiro, Laetitia Teixeira, Rosa Marina Afonso

**Affiliations:** 1 Department of Medical Sciences, Faculty of Health Sciences, University of Beira Interior, Avenida Infante D. Henrique, 6200-506 Covilhã, Portugal; 2 CICS-Health Sciences Research Centre, University of Beira Interior, Covilhã, Portugal; 3 Faculty of Psychology, University of Salamanca, Salamanca, Spain; 4 UNIFAI, Instituto de Ciências Biomédicas Abel Salazar, University of Porto, Porto, Portugal; 5 CINTESIS - Center for Health Technology and Services Research, Faculty of Medicine, University of Porto, Porto, Portugal; 6 Department of Psychology, Faculty of Social and Human Sciences, University of Beira Interior, Covilhã, Portugal

**Keywords:** Centenarians, Cognitive status, Motor function, Disease burden

## Abstract

**Background:**

Centenarians are a growing population in Europe and present significant variability in motor and cognitive functions. The aim of our study was to characterize health status, as well as cognitive and motor functions in a group of Portuguese centenarians. In addition, our study also aimed at analyzing the relationship between cognitive functions and the burden of diseases affecting the elderly.

**Methods:**

Fifty-two centenarians were evaluated using the Mini-Mental State Examination, short version. Walking-related parameters (velocity and time spent in the 3 m walk test), grip strength and number of age-related illnesses were also measured. The relationship between cognitive scores and time spent in the three metre walk test, velocity, grip strength and number of diseases was analysed.

**Results:**

Cognitive scores showed a positive correlation with both handgrip strength and time spent in the three metre walk. In contrast, no association was found between cognitive scores and the presence/absence of disease, walking velocity or number of diseases present.

**Conclusions:**

These results suggest that in centenarians, cognitive functions may be related with motor functions.

## Background

Centenarians are a fascinating population that has increased in numbers since the middle of the last century. Factors such as aging of the general population, improvement in social and medical conditions, genetic and personal characteristics of each elderly individual as well as environmental aspects, may contribute towards the capacity to survive for more than 100 years. It has been estimated that between 2000 and 2050, the number of centenarians will increase more than 16-fold, with around 850,000 centenarians living in the US in 2050, for example [1]. In Portugal, the number of centenarians has almost tripled over the last 10 years from 589 in 2001 [2] to 1526 in 2011 [3] and recent projections from the National Institute of Statistics of Portugal indicate a substantial increase in these numbers in the future.

At present, the full mechanisms associated with reaching 100 years of life or longer are not completely understood. A genetic factor has been suggested in family studies, and many centenarians report very good health into their nineties, having been able to avoid critical periods in their lives. The living environment also seems to play an important part, and Sardinia (Italy), New England (US) and some regions in Japan are examples of places where longevity seems to be a rule for a significant number of people [4].

Health conditions associated with extreme aging are not simple: cognitive decline, muscle weakness and immobilization are present in a large number of very old individuals, frequently in association [5]. There is, however, great heterogeneity regarding these health parameters in near-centenarians and centenarians, with functional capacity ranging from being bedridden to full working capacity. One of the first centenarian studies involving German citizens showed that 20–30% were still completely active, 50% had some type of dementia and 20–30% were bedridden and severely impaired [6]. More recently a study of Chinese centenarians (369 male and 1751 female) showed good self-reported health in 56% of male and 50.6% of female centenarians [7]; in the same study, 51% of the men and 46.4% of the women were still active and 43.3% of men and 25.4% of women still had normal or high cognitive scores. Similar results were reported in the Ipsen survey in France, which showed that 65.2% of centenarian men still had good or very good health, compared with 56.5% of female centenarians. In this study significantly more men than women had normal or high cognitive test results (39.1% versus 17.9%, respectively [8]. In contrast, in the Tokyo centenarian study only 20% of centenarians enjoyed physical and cognitive well being, regardless of their sex [9].

Physical exercise, either aerobic or with an emphasis upon specific motor abilities, has been shown to improve cognition in elderly people [10]. Both types of physical exercise are important to an independent life and previous research showed an association between exercise and cognition in middle aged and elderly individuals [11]. Gait speed is an important factor in old age, and constitutes a predictive indicator of functional capacity in older adults [12]. Handgrip strength was related to cognition in very old subjects: those with the lower values in handgrip were at higher risk of subsequent functional decline and mortality [13]. Furthermore, handgrip strength and walking test results have been shown as prognostic indicators of subsequent mortality in hospitalized elderly people [14]. Several authors have shown an association between handgrip strength and physical and cognitive functionality in old and very old people and also that handgrip strength is an important predictor of functionality in very old people [13–16].

However, it is not known whether handgrip strength (and walking parameters) are related to cognitive functions in centenarians. Our hypothesis is that both parameters are still related even in very old subjects. Thus, this study aims to evaluate a group of centenarians from two regions of Portugal (Oporto and Beira Interior) and to look for any relationship between their cognitive status, their disease burden and some measures of their physical capacity, namely handgrip and walking parameters.

## Methods

### Sample selection and study design

Participants included in this study were elderly Portuguese individuals from two Centenarian Studies: the PT100 Oporto Centenarian Study and the PT100 Beira Interior Centenarian Study [17]. The first step in recruitment of centenarians was to identify and locate all potential participants in each municipality and parish, based upon the census information. The PT 100 team then contacted these centenarians and their families and/or caregivers, in order to collect their sociodemographic and health-related information from parish councils, parishes/churches, nursing homes, institutions, and health care centres. Inclusion criteria included being a centenarian, being able to perform walking speed and handgrip strength tests, as well as being able to provide data for cognitive status assessment. Centenarians that did not have results for the three items were excluded. Of the total of centenarians of the PT100 study (*N* = 241), 43 were excluded because they did not have a score for the MMSE. Out of the remaining 198 centenarians, 146 were excluded because no results were available from the handgrip strength test and/or the walking test. Thus, the final sample of this study included 52 centenarians.

The study was approved by the Ethics Committee of Hospital Sousa Martins, Guarda, Portugal. All centenarians and/or their next of kin or caregivers signed a written informed consent form.

### Assessment of motor functions

Assessment of walking speed was based upon the time centenarians took to walk 3 m at their normal speed. This allowed the indirect evaluation of the velocity a person was able to walk at. Subjects were allowed to rest and self pace as needed while walking along a measured space, in order to obtain their walking velocity [18–20]. Handgrip strength was assessed using a dynamometer (Hand Dynamometer Model Lafayette 78010), and after demonstrating the procedure to the subject, an experience with the non dominant hand was performed three times, with a pause of 10–20 s between each experience in order to avoid muscle fatigue. Results were recorded and the mean was calculated to the nearest Kg. unit. Results were classified in accordance with previously published tables for Lafayette Model 78010 Dynamometer [21].

### Assessment of cognitive status

Cognition was assessed using a shortened version of the Mini Mental State Examination - SMMSE [22, 23]. Following the recommendation by Holtsberg et al [24]., the selected shorter version included only those items that were not biased by sensory impairment which is often prevalent in very old age, resulting in a maximum score of 21 points instead of 30. The short version is frequently used in centenarian people, allowing credible results [24]. The MMSE is one of the most popular tests used for a rapid evaluation of the mental state; it is a rough measure of cognition and indirectly assesses the interest and the ability of the subject to comply with requested tasks and to understand the questions. It is the most widely used cognitive test in very old individuals, since it is quick to perform, is not tiresome, and can be applied in illiterate individuals [25].

### Assessment of age-related ilnesses

Health status was assessed by means of a checklist of common age-related illnesses which included hypertension, heart disease, diabetes, chronic lung disease, gastric ulcer or other serious stomach issues, cirrhosis or other liver problems, kidney diseases, frequent urinary infections, incontinence, prostate problems, problems with vision, problems with hearing, arthritis, osteoporosis, stroke, cancer, pneumonia in the previous five years, according to the lists published previously [26]. Health-related information was given by the centenarians, the centenarians’ informal caregiver, or the centenarian formal caregivers, as appropriate.

### Statistical analysis

Exploratory analysis was performed using univariate analysis. Bivariate analyses were performed using Pearson’s correlation coefficient or Spearman’s correlation coefficient, according to the nature and distribution of the variables involved. Statistical analysis was performed using the Statistical Package for the Social Sciences (SPSS) version 21 and a significance level of 0.05 was used.

## Results

### Sample characteristics

Out of 241 PT100 centenarians, 43 were excluded since sMME results could not be obtained. Out of the remaining 198 centenarians with complete sMME results, 146 had to be excluded due to the fact that handgrip strength and/or walking test results could not be obtained. Thus, a total number of 52 centenarians were evaluated.

Table 1 shows the socio-demographic characteristics of the 52 centenarians. Mean age was 101.2 years (SD = 1.4 years). Most centenarians were women (*n* = 44, 84.6%) and widowed (*n* = 45, 85.5%). Thirty seven (71.2%) centenarians lived in their own homes (community dwelling) and 15 (28.8%) in residential care. Half of the centenarians did not have any schooling (*n* = 26; 51.0%) and only 3 (6.0%) had more than 4 years of schooling. Since only eight men were part of the sample, a sex related analysis was not performed. The median number of diseases was 2.0 (2.0).

**Table 1:** Socio-demographic and health characteristics of the sample (*N* = 52)

	Number	Percent
Gender		
Male	8	15.4
Female	44	84.6
Age, mean (SD)	101.1 (1.3)	
Marital status		
Single	5	9.6
Married/living together	2	3.8
Divorced	0	0.0
Widowed	45	86.5
Education		
No schooling	26	51.0
1–4 years	23	43.0
> 4 years	3	6.0
Severe disease		
Yes	36	69.2
No	16	30.8

### Analysis of motor functions - hand grip strength and walking speed

We first analyzed strength and mobility in this group of centenarians. Hand grip strength showed a mean value of 12.5 kg (SD = 5.9; range 0–27.55). Results regarding hand grip strength are shown in Table 2.

**Table 2:** Hand grip strength frequencies (Kgs)

Strength (Kgs)	N° of centenarians	Percent
0,00–2.30	3	5.4
2.31–4.60	3	5.4
4.61–6.90	2	3.6
6.91–9.20	8	14.4
9.21–12.50	16	28.8
12.51–14,80	7	12.6
14.81–17.10	8	14.4
17.11–19.40	2	3.6
19.41–22.71	2	3.6
22.71–25.00	1	1.8
25.01–27.55	3	5.4

Two subjects (3.6%) did not have any handgrip strength (Kg = 0), five centenarians were able to reach a hand grip strength of more than 20 Kgs, and one subject was able to attain 27.55 kgs.

Results regarding time spent for a 3 m walk are shown in Table 3 and 4.

**Table 3:** Time spent for a 3 m walk in centenarians (in seconds)

Time spent/3 m	Number of subjects
0– < 10 seg	25
10– < 20 seg	22
20– < 30 seg	3
30– < 40 seg	1
40– < 50 seg	1
50– < 60 seg	0
>60 seg	1

**Table 4:** Velocity of the centenarians in the walking test

Velocity (m/s)	Number of centenarians	Percent
0–0,09	2	3.8
0,1–0,19	12	23.1
0,2–0,29	13	25
0,3–0,39	8	15.4
0,4–0,49	7	13.5
0,5–0,59	4	7.7
0,6–0,69	2	3.8
0,7–0,79	1	1.9
0,8–0,89	0	0
0,9–0,99	0	0
1–1,09	2	3.8
1,1……….- 5,99	0	0
6	1	1.9

The mean value of the velocity of the subjects for the three metres test was 0.46 m/s (SD: 0.82m/s; range: 0.045–6.00 m/s). One subject finished the three metre walk in 0.5 s, while six subjects took more than 24 s to perform the same task.

Results transformation allowed us to obtaine velocity values for this group of centenarians (Table 4).

Next, we analyzed whether there was any association between strength and walking speed in the centenarians. There was no linear correlation between handgrip and walking speed (R = -0.083; *p* = 0.560).

### Analysis of cognitive functions

Finally, in terms of their cognitive status, results from the SMMSE showed that 12 centenarians (23; 07%) were able to score more than 16 points, while twenty three (44.2%) had a score of ten or lower points (Table 5).

**Table 5:** Scores for MMSE (short version)

MMSE scores	N° centenarians	%
0–5	8	15.4
6–10	15	28.8
11–15	17	32.7
16–20	10	19.2
21–25	2	3.8

### Analysis of the association between cognitive functions and health status

The association between presence/absence of severe disease and the other variables was performed using the Mann Whitney test (Table 6). For the three variables – handgrip, velocity and SMMSE, no differences between groups with and without disease were found (*p* value <0.5).

**Table 6:** Association between cognitive functions and health status

	Handgrip		Velocity		Time spent		SMMSE	
	R	p	R	p	R	p	R	p
SMMSE	0.331	0.017	0.159	0.260	-0.348	0.012	-	-
Severe Diseases	-0.051	0.721	-0.100	0.479	-	-	0.075	0.599

## Discussion

To the best of our knowledge, this is the first study on physical performance and cognition performed with a sample of Portuguese centenarians. Results showed a significant correlation between cognitive status and some motor function in this group of people older than 100 years (namely handgrip strenght and time spent in the three metre walk). In contrast, no correlation was found between handgrip strength and walking velocity or between physical performance and the presence/absence of severe disease.

The significant correlation that we found between some aspects of physical performance and cognitive scores in centenarians seems important [12, 15]. As individuals grow old, motor abilities are reduced as compared with younger adults. The loss of muscular mass correlates with loss of strength and can be assessed using the handgrip test, which is an indirect measure of health and muscular function [27, 28]. Cognitive changes are also identified in healthy elderly people but, if they are not related to disease, such changes can be compensated. Elderly people achieve cognitive responses which are similar to those seen in younger adults although this seems to occur through a more expanded use of cortical areas, as seen using neuroimaging studies [29, 30]. This expanded utilization of cortical areas can occur with simple motor tasks as well as with more complex motor activities involving coordination, either because old people need to aggregate more motor neurons in different areas or because they need to activate more networks in order to achieve the same result as that obtained by younger adults [31, 32]. In future studies, neuroimaging of centenarian brains with functional MRI may help to clarify this issue.

Handgrip is an useful single marker of frailty and an important predictor of functionality [15, 16]. The relationship found between handgrip and cognitive scores is important, since both items are related to the autonomous functionality of the subjects. A co-dependence of cognition and motor activity has been found in several research studies. For example, the performance in the Trail Making Test, when impaired, is associated with a reduction in walking velocity, particularly with obstacles, in older individuals [11]. Furthermore, walking performance and several neuropsychological tests, including memory, language and executive functions seem to be correlated [33]. It is possible that in centenarians, as in non-centenarian elderly people, motor function and cognition may share some neural networks due to a reduction in neural substrates, and also because cognition is necessary for motor function, or because more extended networks are necessary for both functions. We can also argue that this is probably what conveys the positive effect of physical exercise over neurodegenerative disorders. It has been demonstrated that the increase in complexity of movements and coordination tasks and other types of motor interventions in these patients are associated with an improvement in cognition [10, 32–35]. Different exercise programmes in healthy adults seem to lead to similar cognitive improvements. In a group of older adults both progressive resistance training and multi component training (balance, coordination and resistance) were similarly effective at improving cognition [35], and coordination training and cardiovascular training produced similar positive cognition results in a group of older adults although these types of training activate different areas of the brain [10, 35–37]. On the other hand, several longitudinal studies showed that old people with walking difficulties have a higher risk of developing dementia [38, 39]. Taken together with our results, these studies may suggest that the activation of motor networks of different types involves the activation of cognitive networks.

We could not find any correlation between handgrip strength and time to perform the three metre tests in our sample of centenarians. We could expect that both physical functions would be correlated, since they represent the motor capacity of the subject. However the neural subtract for both characteristics is different: strength seems to be a pure motor function related to the performance of the motor cortex and the peripheral nervous system and muscles, while walking implies a much more sophisticated situation, associating motor cortex, basal ganglia and cerebellum. The absence of correlation observed in our study may thus result from these different neural processes [40].

We would expect centenarians to show some cognitive decline, muscular weakness or immobilization or even a joint decline in these three parameters. Centenarians are survivors of several bouts of diseases but these may present clinically in various ways [7–9, 41]. In our study, about 70% of the individuals had some form of severe disease. However, the ones with severe disease did not show any statistical difference in motor function or cognition when compared with the ones with no disease. This result may be related to the small number of subjects we studied, but it may also be due to the fact that the questionnaire used to obtain information about chronic diseases was very vague, associating information about several disorders but without defining the degree of severity of each one. It is possible that the use of a more detailed inquiry with a gradient of severity could yield a more precise result. It is also possible that, due to the exceptional resilience that being a centenarian represents, disease may not affect the motor and cognitive function in the same way as with younger subjects. Further studies are needed to ascertain this.

Our study had some limitations. Firstly, our strategy was to collect data from the subjects with information on three tests – handgrip, walking and SMMSE; we did not use data from centenarians who, for some reason, did not have one of the three measures. In setting this exclusion criterion, it is possible that we had some form of selection of the more competent people in both motor and cognitive areas. Secondly, although the short version of the MMSE is the most frequent test used in centenarians, due to its simplicity, it is also its simplicity that warrants some degree of caution, because it gives a rough approach to cognitive status.

## Conclusions

Our study found that there is a positive association between motor and cognitive function in centenarians. This suggests that even in very old individuals, an association between motor capacity and cognition is still present.
